# Effect of reproductive ageing on pregnant mouse uterus and cervix

**DOI:** 10.1113/JP273350

**Published:** 2017-02-08

**Authors:** Rima Patel, James D. Moffatt, Evangelia Mourmoura, Luc Demaison, Paul T. Seed, Lucilla Poston, Rachel M. Tribe

**Affiliations:** ^1^ Division of Women's Health, King's College London, Women's Health Academic CentreKing's Health PartnersSt Thomas' HospitalLondonUK; ^2^ Division of Biomedical SciencesSt George's University of LondonLondonUK; ^3^ Université Joseph FourierLaboratoire de Bioénergétique Fondamentale et AppliquéeGrenobleFrance; ^4^ Unité de Nutrition Humaine, INRA, UMR 1019, Clermont UniversitéUniversité d'AuvergneClermont‐FerrandFrance

**Keywords:** cervix, maternal age, mitochondria, myometrium, parturition, smooth muscle, uterus

## Abstract

**Key points:**

- Older pregnant women have a greater risk of operative delivery, still birth and post‐term induction.
- This suggests that maternal age can influence the timing of birth and processes of parturition.
- We have found that increasing maternal age in C57BL/6J mice is associated with prolongation of gestation and length of labour.
- Older pregnant mice also had delayed progesterone withdrawal and impaired myometrial function.
- Uterine ageing and labour dysfunction should be investigated further in older primigravid women.

**Abstract:**

Advanced maternal age (≥35 years) is associated with increased rates of operative delivery, stillbirth and post‐term labour induction. The physiological causes remain uncertain, although impaired myometrial function has been implicated. To investigate the hypothesis that maternal age directly influences successful parturition, we assessed the timing of birth and fetal outcome in pregnant C57BL/6J mice at 3 months (young) and 5 months (intermediate) *vs*. 8 months (older) of age using infrared video recording. Serum progesterone profiles, myometrium and cervix function, and mitochondrial electron transport chain complex enzymatic activities were also examined. Older pregnant mice had a longer mean gestation and labour duration (*P < *0.001), as well as reduced litter size (*P < *0.01) *vs*. 3‐month‐old mice. Older mice did not exhibit the same decline in serum progesterone concentrations as younger mice. Cervical tissues from older mice were more distensible than younger mice (*P < *0.05). Oxytocin receptor and connexin‐43 mRNA expression were reduced in the myometrium from 8‐month‐old *vs*. 3‐month‐old mice (*P < *0.05 and *P < *0.01 respectively) in tandem with more frequent but shorter duration spontaneous myometrial contractions (*P < *0.05) and an attenuated contractile response to oxytocin. Myometrial mitochondrial copy number was reduced in older mice, although there were no age‐induced changes to the enzymatic activities of the mitochondrial electron transport chain complexes. In conclusion, 8‐month‐old mice provide a useful model of reproductive ageing. The present study has identified potential causes of labour dysfunction amenable to investigation in older primigravid women.

## Key points

- Older pregnant women have a greater risk of operative delivery, still birth and post‐term induction.
- This suggests that maternal age can influence the timing of birth and processes of parturition.
- We have found that increasing maternal age in C57BL/6J mice is associated with prolongation of gestation and length of labour.
- Older pregnant mice also had delayed progesterone withdrawal and impaired myometrial function.
- Uterine ageing and labour dysfunction should be investigated further in older primigravid women.

**Abbreviations**
B2M: β_2_ microglobulin
Cx43: connexin‐43
CI: confidence interval
GAPDH: glyceraldehyde 3‐phosphate dehydrogenase
MIT: mean integral tension
MMP2: matrix metalloproteinase‐2
mtDNA: mitochondrial DNA
Mt/N: mitochondrial/nuclear genome ratio
OXTR: oxytocin receptor
PTGS2: prostaglandin‐endoperoxide synthase 2
PSS: physiological saline solution
ROS: reactive oxygen species

## Introduction

Over recent decades, the average age of primigravid mothers in developed countries has increased progressively, with women (including multiparous women) over the age of 35 years comprising a significant proportion of the pregnant population (Office for National Statistics for England and Wales, 2013) (Matthews & Hamilton, 2014). Such trends are accompanied by a simultaneous rise in the incidence of pregnancy complications such as post‐term induction, failure to progress in labour, and postpartum haemorrhage (Ecker *et al*. 2001; Roos *et al*. 2010; Yogev *et al*. 2010). Advanced maternal age is also associated with an increase in Caesarean section and instrumental delivery rates (Smith *et al*. 2008; Ludford *et al*. 2012; Karabulut *et al*. 2013; Herstad *et al*. 2015).

There is limited understanding of the links between maternal age and the processes of the timing of parturition. Spontaneous contraction of isolated human pregnant and non‐pregnant myometrium *ex vivo* is reported to decline with advancing maternal age, with the myometrium from pregnant women exhibiting increased multiphasic contractions (Smith *et al*. 2008; Arrowsmith *et al*. 2012). Other studies have suggested that myometrial tissue responds less effectively to uterotonic agents such as oxytocin or prostaglandins with increasing maternal age (Greenberg *et al*. 2007; Arrowsmith *et al*. 2012), which is supported by the observation that women of older age have a greater probability of requiring oxytocin‐augmentation for the induction of labour (Adashek *et al*. 1993; Main *et al*. 2000).

Age may also influence cervical ripening. A retrospective, cohort study revealed that a maternal age above 30 years was an independent and significant predictor of cervical ripening failure in response to prostaglandin E_2_ (Melamed *et al*. 2010), suggesting an abnormal age‐related timing of cervical ripening or impaired prostaglandin E_2_ responsiveness. A maternal age greater than 35 years has also been implicated as an independent risk factor of ‘failure to progress in labour’ during the first stage of labour, which is normally associated with cervical dilatation and regular myometrial contractions (Sheiner *et al*. 2002).

Studies in rodents may provide further insights into the responisble mechanism and improve upon experimental control and tissue availability compared to the clinical setting, although they have seldom been undertaken in appropriately aged and nulliparous rodents. Holinka *et al*. (1978) suggested that multiparous mice at the extreme limit of reproductive viability (11–12 months) have extended gestations compared to younger multiparous mice (3–7 months), possibly as a result of changes in the maternal progesterone status (Holinka *et al*. 1978). However, the impact of maternal age in nulliparous mice at an age more relevant to older pregnant women (i.e. at the initial stages of reproductive ageing; ∼8 months) was not investigated. In another study, spontaneous myometrial contractility *in vitro* was reduced in tissues from older 24‐week‐old Wistar rats compared to 8‐week‐old animals, although this was neither related to the expression of myometrium contraction associated proteins, nor plasma lipid or pregnancy‐related hormone profiles. There was also no evidence of a delay in timing of delivery (Elmes *et al*. 2015) suggesting that their model does not reflect adequately the situation in older pregnant women. A possible reason for this is that a rat age of 24 weeks is more representative of an 18–20‐year‐old adult rather than a 35‐year‐old (Sengupta, 2013).

The present study aimed to develop a more appropriate mouse model of reproductive ageing to address parallels with older (∼35 years) nulliparous pregnant women and explore the impact on gestational age at parturition, as well as uterine and cervical function. We hypothesized that the timing/initiation of labour would be prolonged in older dams as a consequence of a delay in parturition signals affecting the physiological ripening of the cervix and the activation of myometrium. We also explored the notion that the ability of the myometrium to contract at term would deteriorate in older mothers as a result of age‐related mitochondrial dysfunction.

## Methods

### Ethical approval

Institutional Animal Welfare Committee approval was not required because no regulated procedures were used for the present study. No animal was anaesthetized or underwent any surgical procedure. All animals were treated and killed in accordance with the Animals (Scientific Procedures) Act 1986 guidelines and the experimental details conform with the animal ethics checklist (Grundy, 2015).

### Animals

C57BL/6J mice (weighing 21.5–30.5 g, aged 3–8 months; Charles River Laboratories, Margate, UK) were maintained under controlled conditions (25 °C, 12:12 h light/dark cycle) and received water and standard chow diet *ad libitum*. Females (all ages) were mated with C57BL/6J males (aged 3–4 months; Charles River Laboratories). Conception was determined by the presence of a vaginal plug (day 0 of gestation). A total of 510 mice were used for the present study. This number comprised non‐pregnant 3‐month‐old (*n = *60), 5‐month‐old (*n = *50) and 8‐month‐old (*n = *60) mice; late pregnant 3‐month‐old (*n = *90), 5‐month‐old (*n = *40) and 8‐month‐old (*n = *110) mice; and full‐term 3‐month‐old (*n = *8) and 8‐month‐old (*n = *8) mice; as well as 84 pups born to the full‐term mice.

### Development of the mouse model of advanced maternal age

Three age groups of female mice were selected for experimentation; all were capable of reproduction (3, 5 and 8 months of age). This was informed by previous studies of the oestrous cycle in ageing C57BL/6J mice (Nelson *et al*. 1982; Felicio *et al*. 1984), which reported that peak cycling occurred at 3 to 5 months of age but that, by 9 months, normal cycling was disrupted and average cycle lengths became progressively longer, indicative of reproductive ageing. Virgin C57BL/6J mice at 8 months of age were used to model human nulliparous advanced maternal age. The 3‐month‐old mice acted as controls, assuming this to be a period of maximal reproductive potential. The 5‐month‐old mice represented an intermediate age group for some experiments. Where appropriate, experimental techniques were repeated with age matched non‐pregnant female mice.

### Tissue collection

Mice were killed by cervical dislocation and exsanguination, in accordance with Schedule 1 of The Animals (Scientific Procedures) Act 1986. To control for changes to uterine smooth muscle during the oestrous cycle, all non‐pregnant tissues were collected in the oestrous stage of the cycle, as confirmed by the presence of large cornified epithelial cells with very few or no visible nuclei in vaginal fluid collected by daily vaginal smearing.

Myometrium and cervixes from time mated late pregnant mice were collected on day 18 of gestation and blood samples for progesterone analyses taken between days 15 and 19 of gestation. Blood samples were immediately collected by cardiac puncture. Following centrifugation (10 min at 13 000 *g*), serum was aspirated and stored at –80 °C until analysis.

Uterine horns and cervices were dissected and placentas and fetuses were removed. Fetuses were killed by destruction of the brain and decapitation, in accordance with Schedule 1. Mouse uterine horns were cut longitudinally down the midline, and the endometrium was gently removed with a cotton bud. Small, full thickness myometrium strips were dissected longitudinally with the fibre structure and mounted vertically in the organ bath.

Uterine and cervical tissues were either immediately snap frozen in liquid nitrogen for DNA extraction and protein isolation, placed into RNAlater (Ambion, Warrington, UK) in accordance with the manufacturer's instructions for RNA extraction and stored at −80 °C, or transferred to ice‐cold PBS (Sigma‐Aldrich, Poole, UK) for isometric tension recording. Cervical biopsies for histological analysis were fixed by overnight immersion in 10% (v/v) formal saline solution (formaldehyde solution 10% v/v in 0.9% NaCl solution; Fisher Scientific, Loughborough, UK) and then embedded in paraffin. Non‐pregnant uterine and cervical tissues were collected and processed similarly.

### Serum progesterone assay

Serum progesterone concentrations from primiparous mice through late gestation (*n = *5–8) were determined using a progesterone enzyme‐linked immunosorbent assay kit (Enzo Life Sciences, Exeter, UK).

### RNA isolation and cDNA synthesis

Myometrium (30 mg) was homogenized (TissueLyser; Qiagen, Manchester, UK) and total RNA was extracted from the lysate (RNeasy mini kit; Qiagen) in accordance with the manufacturer's instructions. RNA sample quality and concentrations were verified by gel electrophoresis and spectrophotometric analysis using a NanoDrop ND‐1000 spectrophotometer (Labtech International Ltd, Uckfield, UK). RNA (500 ng) was used to synthesize cDNA using 0.25 μg of random hexanucleotide primers (Promega, Southampton, UK) and 200 IU of Superscript III (Invitrogen, Paisley, UK).

### DNA isolation

Total genomic DNA was extracted from 25 mg of myometrial tissue. Tissue was homogenized in 160 μl of PBS (Sigma‐Aldrich). DNA was extracted from the lysate using the ReliaPrep gDNA Purification kit (Promega) in accordance with the manufacturer's instructions. DNA was quantified (ng μl^−1^) and the DNA integrity was checked (NanoDrop ND‐1000 spectrophotometer). Dilution was standardized to 50 ng μl^−1^ in a volume of 100 μl using RNase/DNase free water (Qiagen) and samples were sonicated for 10 min (Pulsatron 55; Kerry Ultrasonics Ltd, Hitchin, UK) to shear the DNA prior to mitochondrial/nuclear genome ratio (Mt/N) ratio determination by a quantitative real‐time PCR.

### Quantitative real‐time PCR

A quantitative real‐time PCR was performed as described by Bustin *et al*. (2008). SYBR green chemistry (2x QuantiFAST SYBR green; Qiagen) was performed on a RotorGene 6000 (Qiagen) using the primers listed in Table 1. Forward and reverse primers were optimized to be used at working concentrations of 300 nm in a total reaction volume of 10 μl. A pre‐PCR cycle was run for 5 min at 95 °C followed by 42 cycles of 95 °C for a 10 s denaturation step, followed by 60 °C for a 30 s combined annealing/extension step. Test samples (2 μl of cDNA) were run in duplicate in parallel with cDNA standards of known gene copy number abundance (10^8^ to 10^1^ copies). Melt‐curve analysis was performed to confirm the presence of one single product and non‐template controls were run to assess genomic DNA contamination. Qunatification cycle (also referred to as cycle threshold) values were used for analysis and abundance data were obtained for test samples by the generation of a standard curve based on the quantified cDNA. Quantification data for the genes of interest were expressed relative to the most stable reference genes from a panel of 3 [glyceraldehyde 3‐phosphate dehydrogenase (GAPDH), β‐actin and β_2_ microglobulin (B2M)], using GeNorm software (Vandesompele *et al*., 2002).

**Table 1:** Primer sequences used in quantitative PCR

Gene	Primer sequences
mouse GAPDH (glyceraldehyde‐3‐phosphate dehydrogenase) (accession number: NM_001001303)	Forward primer: 5′‐TTGATGGCAACAATCTCCAC‐3′ Reverse primer: 5′‐CGTCCCGTAGACAAAATGGT‐3′
mouse B2M (beta‐2 microglobulin) (accession number: NM_009735)	Forward primer: 5′‐TTCAGTATGTTCGGCTTCCC‐3′ Reverse primer: 5′‐TGGTGCTTGTCTCACTGACC‐3′
mouse β‐ actin (accession number: NM_007393)	Forward primer: 5′‐ATGGAGGGGAATACAGCCC‐3′ Reverse primer: 5′‐TTCTTTGCAGCTCCTTCGTT‐3′
mouse OTR (oxytocin receptor) (accession number: NM_001081147.1)	Forward primer: 5′‐GTGCAGATGTGGAGCGTCT‐3′ Reverse primer: 5′‐GTTGAGGCTGGCCAAGAG‐3′
mouse Cx43 (connexin 43) (accession number: X61576.1)	Forward primer: 5′‐GTGCCGGCTTCACTTTCA‐3′ Reverse primer: 5′‐GGAGTAGGCTTGGACCTTGTC‐3′
mouse PTGS2 (Prostaglandin‐endoperoxide synthase 2) (accession number: NM_011198.3)	Forward primer: 5′‐GGGAGTCTGGAACATTGTGAA‐3′ Reverse primer: 5′‐GTGCACATTGTAAGTAGGTGGACT‐3′

### Infrared video camera recording of mouse parturition

Primiparous late pregnant (day 17 of gestation onwards, 3 and 8 months of age, *n = *8 for each) females were singly housed. Females were monitored continuously using infrared video cameras (600TVL Eyeball Dome; Qvis, Austin, TX, USA) from the appearance of the first pup until the completion of parturition. Minimal nesting bedding was provided (Enviro‐dri; Shepherd Speciality Papers, Watertown, TN, USA) to prevent the camera view being obscured. Mice were maintained under controlled conditions (25 °C, 12:12 h light/dark cycle: 07.00–19.00 h) with access to standard chow and water *ad libitum*. Cameras were connected to a Zeus Lite HDMI LX 4 Channel Full D1 Networked CCTV Recorder DVR 1TB Hard Drive (Qvis), which recorded continuously with the tracked time and date. Litter size, live pup weights and any pup mortality were recorded. Mice that failed to deliver after 21 days were culled and the uteri were dissected to confirm pregnancy loss. The precise time of birth was determined from the video recording by the appearance and complete delivery of the first pup, and gestation length calculated (days). Total parturition duration (h) was determined as the time between the complete delivery of the first pup to the complete delivery of the final pup.

### *Ex vivo* isometric tension measurements of mouse myometrium

Myometrial muscle strips (∼5 × 2 × 2 mm) were dissected from mouse uterine horns from non‐pregnant females in oestrous or late pregnant dams (gestation day 18). Strips were mounted in a 10 ml of organ bath chamber (Panlab 8 Chamber Organ Bath System; ADInstruments, Oxford, UK) and maintained in oxygenated physiological salt solution (PSS) (in mm): 119 NaCl, 4.7 KCl, 1.17 MgSO_4_, 25 NaHCO_3_, 1.18 KH_2_PO_4_, 0.025 EDTA, 6 glucose and 2.5 CaCl_2_; 37 °C, 95% air–5% CO_2_; pH 7.4; BOC Gases, Guildford, UK). A resting tension of 29 mN (2× slack length) was applied before an equilibration period (45 min) to establish spontaneous contractions. After 10 min of baseline measurement, spontaneous contractile activity (minimum 1 h) and the response to the contractile agonist oxytocin were evaluated (10^−12^ to 10^−7^ m oxytocin concentration response curve; Syntocinon; Alliance Pharmaceuticals, Chippenham, UK). The contractile capacity of each myometrial tissue strip in response to a depolarizing K^+^ solution (PSS with 60 mm KCl substituted for NaCl) was measured after recording the spontaneous contractility.

All data were recorded and analysed using LabChart, version 6 (ADInstruments). Contractile periods were assessed using mean integral tension (MIT; the sum of the contraction integrals divided by the duration of the period assessed and then corrected for myometrial active tissue wet weight, expressed as mN·s g^–1^). Contraction force was calculated as the mean amplitude of contractions over the assessment period. Contraction frequency was expressed as contractions s^–1^, and the duration of a contraction as the time (s) between start and end. Because oxytocin dose–response curves were not sigmoidal, EC_50_ and *E*_max_ values could not be calculated, the slope of the linear response across the concentration range was analysed.

### *Ex vivo* tensile strength measurements of mouse cervix

Cervical tensile strength was measured as described previously (Read *et al*. 2007). Intact cervices from non‐pregnant in oestrous or late pregnant (day 18) females were dissected and all vaginal tissue was removed (*n = *6–9). The preparation was mounted in a 10 ml organ bath chamber in oxygenated PSS (37 °C). Cervices were initially held at a slack length (at which a further 0.25 mm stretch led to an increase in passive tension) and equilibrated for 15 min. The inner diameter (resting diameter of cervical os) was measured as 0 mm stretch and the os was then isometrically stretched incrementally (1 mm every 2 min) until tension plateaued or, infrequently, when the tissue snapped/tore. The force required to distend the cervix and the tension exerted by the stretched tissue were analysed, force–strain curves generated and the slope of the linear component measured as a gauge of tissue elasticity/stiffness.

### Histological studies in mouse cervix

Whole intact cervical samples from non‐pregnant females in oestrous or late pregnant (day 18) were fixed overnight [10% (v/v) formaldehyde solution in 0.9% NaCl solution; Fisher Scientific], followed by automated processing (TP1020 tissue processor; Leica Biosystems, Milton Keynes, UK) and wax embedding (EG1150 heated paraffin embedding module; Leica Biosystems). Paraffin wax sections (5–10 μm longitudinally through cervical samples) were mounted on SuperFrost Plus slides (VWR International Ltd, Lutterworth, UK). Masson's Trichrome Kit (Accustain® Trichrome stains; Sigma‐Aldrich) was used to identify structures and detect collagen (blue stain), including a positive control section (Trichrome TISSUE‐TROL; Sigma‐Aldrich). Images (10× magnification of original) were taken using AZ100 multizoom microscope. Collagen content (area of blue stain) in each cervical section was calculated (% of total section area, binary units; NIS‐Elements, version 4.0; Nikon Instruments Europe, Amsterdam, The Netherlands). The identities of the sections were unknown to the observer.

Matrix metalloproteinase‐2 (MMP2) expression was localized by immunostaining (ImmunoCruz rabbit LSAB Staining System; Santa Cruz Biotechnology, Heidelberg, Germany). Cervical sections were deparaffinized, immersed in 10 mm Tris‐EDTA buffer (10 mm Tris Base, 1 mm EDTA, 0.05% Tween 20, pH 8.0) and heated at highest power for 10 min in a microwave (antigen retrieval). Sections were incubated with MMP2 primary antibody (dilution 1:500; Anti‐MMP2 ab37150; Abcam, Milton, UK) overnight at room temperature in a humidified chamber and counterstained with haematoxylin (Gill formulation number 2; Sigma‐Aldrich). Images were taken at 10× magnification of original (AZ100 multizoom microscope and NIS‐Elements, version 4.0; Nikon Instruments Europe). Brown staining was quantified using ImageScope (Aperio Technologies Ltd, Ascot, UK). The percentage of positive labelled cells per 10× magnification field was determined using the ‘positive pixel count’ function. Results were expressed as ‘positivity’, accounting for the number of positive pixels and the intensity of staining. The identities of the sections were unknown to the observer.

### Mitochondrial DNA (mtDNA) copy number in mouse myometrium

Genomic DNA was extracted from mouse myometrium from non‐pregnant females in oestrous and late pregnant (day 18) mice as described (*n = *8). A quantitative real‐time PCR was performed for the primers listed in Table 2 (Malik *et al*., 2016). Data were expressed as the Mt/N ratio: the quantitative real‐time PCR derived copy number for the mouse mitochondrial genome relative to the mouse B2M copy number (Malik *et al*. 2011; Malik *et al*. 2016).

**Table 2:** Primer sequences used to determine mitochondrial copy number

Gene	Primer sequences
mouse mitochondrion complete genome (accession number: NC_005089.1)	Forward primer: 5′‐CTAGAAACCCCGAAACCAAA‐3′ Reverse primer: 5′‐CCAGCTATCACCAAGCTCGT‐3′
mouse B2M (accession number: NC_000068.8)	Forward primer: 5′‐ATGGGAAGCCGAACATACTG‐3′ Reverse primer: 5′‐CAGTCTCAGTGGGGGTGAAT‐3′

### Mitochondrial electron transport chain enzymatic activities in mouse myometrium

Myometrial samples (non‐pregnant in oestrous and late pregnant gestation day 18, 50 mg, *n = *8) were homogenized [4 °C in 1:9 (w/v) 100 mm potassium phosphate (K_2_HPO_4_· 3· H_2_O) buffer; pH 7.4] and centrifuged (5 min at 1500 *g* and 4°C). Supernatants were stored at −80°C until required. Enzymatic activities of mitochondrial respiratory chain complexes: NADH‐ubiquinone oxydo‐reductase (complex I), succinate‐ubiquinone oxydo‐reductase (complex II), ubiquinol cytochrome *c* reductase (complex III) and citrate synthase were assayed using a spectrophotometer (Uvikon 941; Kontron Instruments, Chichester, UK) as described previously (Mourmoura *et al*. 2011).

Protein content in homogenates was determined via the bicinchoninic acid method using a commercially available kit (Thermo Scientific, Rockford, IL, USA). The activities of the electron transport chain complexes were expressed in units mg^–1^ homogenate protein content. To evaluate myometrial mitochondrial density, citrate synthase activity was adjusted for protein content.

### Statistical analysis

Student's *t* test was used to assess data between two groups. One‐way ANOVA was used followed by an all pairwise multiple comparison (Tukey's test) when comparing three or more groups (GraphPad Prism, version 5.0; GraphPad Software Inc., San Diego, CA, USA). The comparisons presented are between pregnant and non‐pregnant animals at the same age and between animals at different ages with the same pregnancy status.

Data are expressed as the mean ± SEM. *P < *0.05 was considered statistically significant; *n* is the number of animals or tissues used per test group. Linear regression analyses were carried out using Stata, version 11.2 (StataCorp, College Station, TX, USA) and *P < *0.05 was considered statistically significant. Pup weight was calculated as an average per litter. For assessment of myometrium spontaneous contractile activity, when there was more than one tissue strip per animal, the average value across all related strips was used. For assessment of oxytocin augmented concentration responses, the rate of change in MIT (slope) in response to increasing concentrations of oxytocin was calculated for each myometrium tissue strip and the results compared generalized least squares regression with robust standard errors clustered by animal.

Power calculations were carried out based on published data. Animal numbers were calculated to give a minimum of 80% power to detect a minimum of 20% difference between groups at *P < *0.05.

## Results

### Parturition in mice of advancing reproductive age

**Figure 1:**
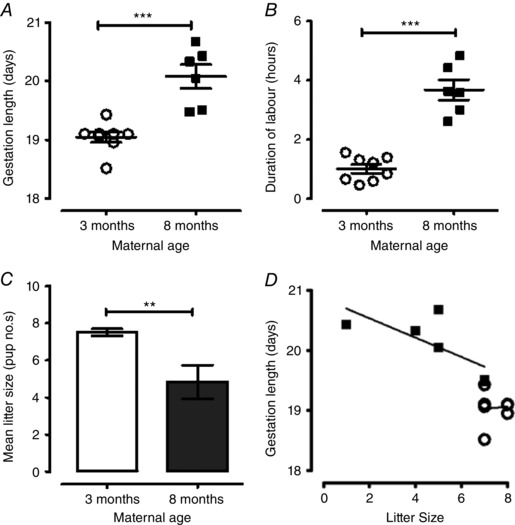
Influence of maternal age on gestation length, labour duration and litter size *A*, scatter graph presenting individual gestation lengths in 3‐month‐old (*n = *8, open circles) and 8‐month‐old (*n = *6, closed squares) pregnant mice. Gestation was longer in 8‐month‐old mice (^***^ *P < *0.001). *B*, scatter graph presenting individual labour durations in 3‐month‐old (*n = *8, open circles) and 8‐month‐old (*n = *6, closed squares) pregnant mice. Parturition was longer in 8‐month‐old mice (^***^ *P < *0.001). Horizontal bars indicate means and error bars indicate the SEM in both (*A*) and (*B*). *C*, litter size for 3‐month‐old (*n = *8, open bar) and 8‐month‐old (*n = *6, closed dark grey bar) pregnant mice. Litter size was reduced in 8‐month‐old mice (^**^ *P < *0.01). Data expressed as the mean* ± *SEM. Data were analysed using Student's *t* test for (*A*), (*B*) and (*C*). *D*, scatter plot and linear regression lines illustrating the relationship between gestation length and litter size in 3‐month‐old (*n = *8, open circles) and 8‐month‐old (*n = *6, closed squares) pregnant mice. There was no significant influence of litter size on gestation length (3‐month slope: 0.04* ± *0.19, *r* ^2^ = 0.007, *P = *0.848; 8‐month slope: –0.16* ± *0.08, *r* ^2^ = 0.523, *P = *0.104).

Eight mice per group were monitored in pregnancy and at delivery. Of the 8‐month‐old mice, only six delivered at term and 25% failed to maintain pregnancy (confirmed by uterine dissection post day 21). All mice, regardless of age group, were confirmed to be pregnant on gestation day 15, although 63% of the older mice had some degree of intrauterine fetal loss and/or fetal resorption. All 3‐month‐old mice delivered at term, with no fetal loss. Older 8‐month‐old mice demonstrated increased gestational length (20.1 ± 0.2 days, *n = *6 *vs*. 19.1 ± 0.1 days for 3‐month‐old mice, *n = *8; *P < *0.001) (Fig. 1 *A*). Eight‐month‐old pregnant mice also had longer labour duration (3.7 ± 0.3 hours, *n = *6 *vs*. 1.0 ± 0.2 for 3‐month‐old mice, *n = *8; *P < *0.001) (Fig. 1 *B*) and reduced litter size (4.8 ± 0.9 pups, *n = *6 litters *vs*. 7.5 ± 0.2 pups for 3‐month‐old mice; *n = *8 litters; *P < *0.01) (Fig. 1 *C*). Reproductive age had no effect on average pup weight per litter; 8‐month‐old mice (1.59 ± 0.07 g, 29 pups from *n = *6 litters *vs*. 1.54 ± 0.04 g, 60 pups from *n = *8 litters).

Linear regression analyses were undertaken to determine whether the prolongation of gestation and duration of labour in older mice could be explained not only by an influence of maternal age, but also mechanistically because of the reduction in litter size. The impact of older age on gestation length was 1.03 days [95% confidence interval (CI) = 0.59–1.47; *P < *0.001], which remained significant after adjustment for litter size. The effect of maternal age on gestation length was 0.64 days (95% CI = 0.12–1.16; *P < *0.05). Litter size had no significant influence on gestation length for either 3‐ or 8‐month‐old mice (3‐month slope: 0.04 ± 0.19; *r* ^2^ = 0.007, *P = *0.848; 8‐month slope: –0.16* ± *0.08; *r* ^2^ = 0.523, *P = *0.104) (Fig. 1 *D*). The impact of maternal age on labour duration was 2.67 h (95% CI = 1.93–3.41; *P < *0.001) after adjustment for litter size; the effect of maternal age on labour duration was 1.77 h (95% CI = 1.14–2.39; *P < *0.001).

### Progesterone measurements in late gestation

**Figure 2:**
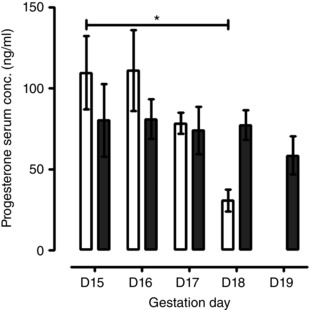
Effect of maternal age on serum progesterone concentration Serum progesterone concentrations through gestation days 15 to 19 (D15–19) for 3‐month‐old (*n = *8 for each day, open bars) and 8‐month‐old (*n = *5–8 for each day, closed dark grey bars) pregnant mice. The 3‐month‐old mice had a significant reduction in serum progesterone concentration from day 15 of gestation to day 18 (^*^ *P < *0.05); however, serum progesterone concentrations were similar throughout gestation (D15–19) for older mice. Data were analysed using ANOVA followed by an all pairwise multiple comparison Tukey's test and are presented as the mean* ± *SEM.

The serum progesterone profile differed between 3‐ and 8‐month‐old mice; in 3‐month‐old mice, the progesterone concentration fell between gestation days 15 and 18 (109.5* ± *22.55 ng ml^−1^ *vs*. 30.5* ± *6.59; *P < *0.01) (Fig. 2), whereas this decline was not observed in older mice between gestation days 15 and 19 (80.9* ± *12.13 to 58.4* ± *11.57 ng ml^−1^) (Fig. 2).

### Effect of reproductive ageing on cervical distensibility

**Figure 3:**
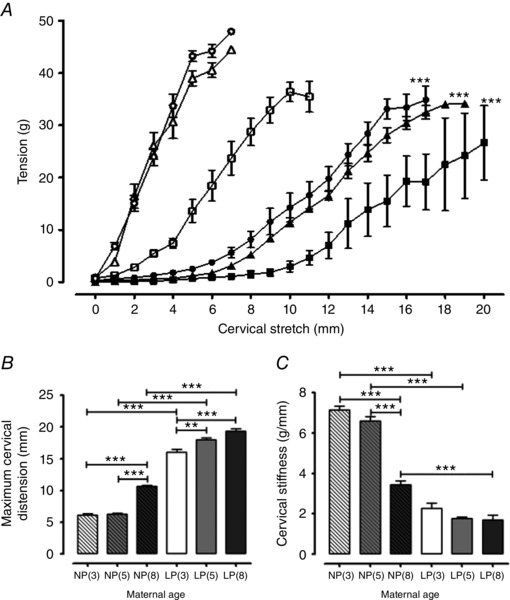
The impact of maternal age on cervical distension and stiffness in non‐pregnant and late pregnant tissues *A*, tension generated by the cervix is plotted as a function of incremental stretch of the cervical os diameter as stress–strain curves. Tissues obtained from non‐pregnant 3‐month‐old (*n = *6, open circles), 5‐month‐old (*n = *8, open triangles) and 8‐month‐old (*n = *8, open squares) mice, as well as late pregnant 3‐month‐old (*n = *9, closed circles), 5‐month‐old (*n = *6, closed triangles) and 8‐month‐old (*n = *6, closed squares) mice. Cervical stretch (distension) means were different between non‐pregnant compared to late pregnant mice of all three age groups (^***^ *P < *0.001). Cervices obtained from non‐pregnant 3‐month‐old [NP(3), *n = *6, open bar with diagonal lines], 5‐month‐old [NP(5), *n = *8, closed grey bar with diagonal lines] and 8‐month‐old [NP(8), *n = *8, closed dark grey bar with diagonal lines] mice, as well as late pregnant 3‐month‐old [LP(3), *n = *9, open bar], 5‐month‐old [LP(5), *n = *6, closed grey bar] and 8‐month‐old [LP(8), *n = *6, closed dark grey bar] mice for (*B*) and (*C*). *B*, transition from a non‐pregnant to pregnant state, significantly increased maximal cervical distension in all three age groups (^***^ *P < *0.001). Advance in age from 3 to 8 months and 5 to 8 months in non‐pregnant mice, as well as from 3 to 5 to 8 months in late pregnant mice, also significantly increased maximal cervical distension (^**^ *P < *0.01 and ^***^ *P < *0.001). *C*, cervical stiffness as determined by the slope of cervical distension stress–strain curves. Transition from a non‐pregnant to pregnant state, significantly reduced cervical stiffness in 3‐, 5‐ and 8‐month‐old mice (^***^ *P < *0.001). Advance in age from 3 to 8 months and 5 to 8 months in non‐pregnant mice also significantly reduced cervical stiffness (^***^ *P < *0.001); however, an advance in age did not change cervical stiffness in tissues from late pregnant mice. Data were analysed using ANOVA followed by an all pairwise multiple comparison Tukey's test and are presented as the mean* ± *SEM for (*A*), (*B*) and (*C*).

Maximal cervical distension (increase in cervical os diameter) was similar in non‐pregnant mice aged 3 and 5 months but, at 8 months, was increased 1.7‐fold compared to both 3‐month‐old (10.6* ± *0.2 mm, *n = *8 *vs*. 6.2* ± *0.2 mm, *n = *6; *P < *0.001) and 5‐month‐old (6.3* ± *0.2 mm, *n = *8; *P < *0.001) non‐pregnant mice (Fig. 3 *A* and *B*). In late pregnancy, cervices from 8‐month‐old mice were more distensible compared to the 3‐month‐old mice (19.3* ± *0.4 mm, *n = *6 *vs*. 16.0* ± *0.5 mm, *n = *9; *P < *0.001) and those of the 5‐month‐old mice more distensible than 3‐month‐old animals (18.0* ± *0.3 mm, *n = *6; *P < *0.01) (Fig. 3 *A* and *B*).

Maximum distension was greater in mice of all ages in late pregnancy compared to non‐pregnant mice, implying that cervical softening had occurred in all groups by day 18 of gestation. The maximal distension for late pregnant cervical tissues compared to maternal age matched non‐pregnant animals was 2.59‐, 2.88‐ and 1.82‐fold higher for 3‐, 5‐ and 8‐month‐old mice, respectively (*n = *6–9; *P < *0.0001 for all) (Fig. 3 *A* and *B*).

Cervical stiffness was determined as the slope of cervical distension stress–strain curves shown in Fig. 3 *A*. There was no difference in cervical stiffness between non‐pregnant 3‐ and 5‐month‐old mice (7.1* ± *0.2 g mm^−1^, *n = *6; 6.6 0.2 g mm^−1^, *n = *8, respectively) but stiffness at both ages was approximately twice that at 8 months (3.4* ± *0.2 g mm^−1^, *n = *8) (*P < *0.001 for both comparisons) (Fig. 3 *C*). Slopes of the cervical distension stress–strain curves for all ages of late pregnant mice were similar and all were lower than age matched non‐pregnant animals by a factor of 0.32, 0.27 and 0.49 for 3‐, 5‐ and 8‐month‐old mice, respectively (*n = *6–9; *P < *0.0001 for all) (Fig. 3 *C*).

### Determination of collagen in cervical tissues

**Figure 4:**
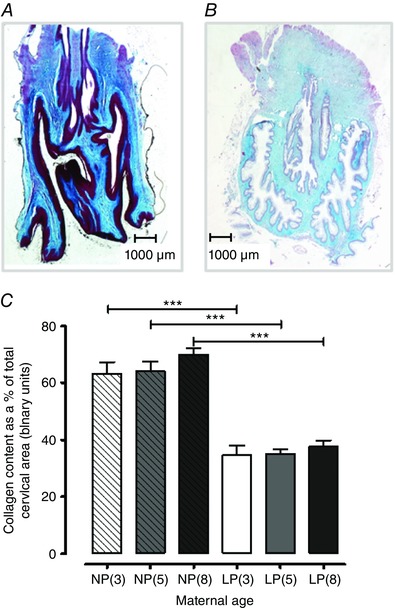
Collagen content is lower in cervical tissues from late pregnant mice compared to non‐pregnant mice Representative images of Masson's trichrome staining of collagen in cervical tissues from 8‐month‐old non‐pregnant (*A*) and late pregnant (*B*) mice. Collagen staining is noticeably weaker in the late pregnant cervix. Original magnification 10×. *C*, percentage of collagen content in cervical tissues obtained from non‐pregnant 3‐month‐old [NP(3), *n = *6, open bar with diagonal lines], 5‐month‐old [NP(5), *n = *6, closed grey bar with diagonal lines] and 8‐month‐old [NP(8), *n = *6, closed dark grey bar with diagonal lines] mice, as well as late pregnant 3‐month‐old [LP(3), *n = *6, open bar], 5‐month‐old [LP(5), *n = *6, closed grey bar] and 8‐month‐old [LP(8), *n = *6, closed dark grey bar] mice. Transition from a non‐pregnant to pregnant state significantly reduced cervical collagen content in 3‐, 5‐ and 8‐month‐old mice (^***^ *P < *0.001). Data were analysed using ANOVA followed by an all pairwise multiple comparison Tukey's test and are presented as the mean* ± *SEM. [Color figure can be viewed at wileyonlinelibrary.com]

Masson's trichrome blue staining (expressed as a percentage of the total cervical area) was similar between the three age groups in the non‐pregnant and late pregnant cervical tissues. The transition from non‐pregnant to late pregnancy in all age groups was associated with reduced cervical collagen content; a 28% decline in 3‐month‐old mice compared to age matched non‐pregnant animals (63.0* ± *4.2% to 34.6* ± *3.5%; *P < *0.001), a 29% reduction in 5‐month‐old mice (64.1* ± *3.4% to 34.9* ± *1.7%; *P < *0.001) and a 32% reduction for 8‐month‐old mice (69.8* ± *2.4% to 37.8* ± *2.1%; *P < *0.001) (*n = *6 for all groups) (Fig. 4 *C*). This was indicated by weaker and sparser blue staining, as shown in Fig. 4 *A* and *B*.

### Expression of MMP2 in cervical tissues

**Figure 5:**
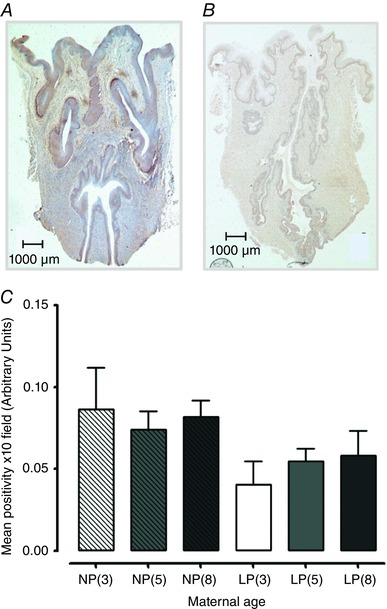
Expression of MMP2 is not significantly altered by the effect of age in cervical tissues from both non‐pregnant and late pregnant mice Representative images of immunohistochemical staining of MMP2 in cervical tissues from 8‐month‐old non‐pregnant (*A*) and late pregnant (*B*) mice. *C*, expression of MMP2 in cervical tissues obtained from non‐pregnant 3‐month‐old [NP(3), *n = *6, open bar with diagonal lines], 5‐month‐old [NP(5), *n = *6, closed grey bar with diagonal lines] and 8‐month‐old [NP(8), *n = *6, closed dark grey bar with diagonal lines] mice, as well as late pregnant 3‐month‐old [LP(3), *n = *6, open bar], 5‐month‐old [LP(5), *n = *6, closed grey bar] and 8‐month‐old [LP(8), *n = *6, closed dark grey bar] mice. Comparisons across all groups did not reach significance. Data were analysed using ANOVA followed by all pairwise multiple comparison Tukey's test and are presented as the mean* ± *SEM arbitrary positivity score. [Color figure can be viewed at wileyonlinelibrary.com]

MMP2 staining was localized in the columnar and squamous epithelium, as well as the stroma, in both non‐pregnant and late pregnant cervical tissues for all three ages. MMP2 protein expression (mean ± SEM arbitrary units of positivity) was not significantly different between the three age groups in non‐pregnant or late pregnant cervical tissues. MMP2 expression was 0.086 ± 0.025, 0.074 ± 0.011 and 0.082 ± 0.010 arbitrary units in cervical tissues from non‐pregnant 3‐, 5‐ and 8‐month‐old mice, respectively. Expression was 0.040 ± 0.015, 0.055 ± 0.007 and 0.058 ± 0.015 arbitrary units in cervical tissues from late pregnant 3‐, 5‐ and 8‐month‐old mice, respectively (Fig. 5; representative staining).

### Contractile‐associated protein gene expression analysis in late pregnant myometrium

**Figure 6:**
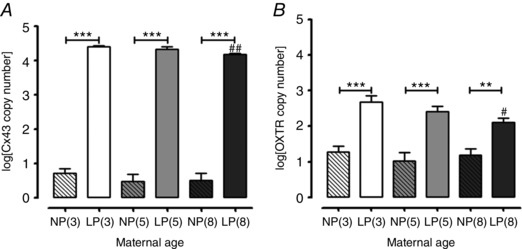
Effect of maternal age on mRNA expression of Cx43 and OXTR in the myometrium from non‐pregnant and late pregnant mice Myometrium was obtained from non‐pregnant 3‐month‐old [NP(3), *n = *8, open bars with diagonal lines], late pregnant 3‐month‐old [LP(3), *n = *8, open bars], non‐pregnant 5‐month‐old [NP(5), *n = *8, closed grey bars with diagonal lines], late pregnant 5‐month‐old [LP(5), *n = *8, closed grey bars], non‐pregnant 8‐month‐old [NP(8), *n = *8, closed dark grey bars with diagonal lines] and late pregnant 8‐month‐old [LP(8), *n = *8, closed dark grey bars] mice for both (*A*) and (*B*). *A*, expression of connexin‐43 was increased in late pregnant myometrium of all age groups compared to non‐pregnant mice (^***^ *P < *0.001) and lower in late pregnant myometrium from 8‐month‐old mice compared to late pregnant 3‐month‐old mice (^##^ *P < *0.01). *B*, expression of oxytocin receptor was significantly increased in late pregnant myometrium of all age groups compared to non‐pregnant mice (^***^ *P < *0.001, ^**^ *P < *0.01) and lower in late pregnant myometrium from 8‐month‐old mice compared to late pregnant 3‐month‐old mice (^#^ *P < *0.05). Data were analysed using ANOVA followed by an all pairwise multiple comparison Tukey's test and are expressed as log(mean of copy number)* ± *SEM normalized to reference genes B2M and GAPDH.

Prostaglandin endoperoxide synthase 2 (PTGS2) mRNA expression was similar in non‐pregnant and late pregnant myometrium (*n = *8 per group) and there were no age‐related differences in expression in the myometrium from both groups. Connexin 43 (Cx43) mRNA expression was reduced in late pregnant myometrium from 8‐month‐old [4.18* ± *0.03 log(Cx43 copy number)] compared to 3‐month‐old (4.40* ± *0.03; *P < *0.01, *n = *8 for both groups) mice (Fig. 6 *A*). There were no other age‐related differences in expression across groups. Cx43 expression was low in non‐pregnant myometrium from all age groups compared to late pregnancy, which was associated with increased expression in mice of all ages (6.16‐, 9.12‐ and 8.17‐ fold change for 3‐, 5‐ and 8‐month‐old mice, respectively, compared to non‐pregnant tissues; *P < *0.001, *n = *8 for all groups) (Fig. 6 *A*).

Myometrial expression of oxytocin receptor (OXTR) was reduced in 8‐month‐old late pregnant mice [2.67* ± *0.19 log(OXTR copy number)] compared to younger mice (3 month; 2.11* ± *0.12; *P < *0.05, *n = *8 for both groups). There were no other age‐induced differences in OXTR expression levels across groups. Late pregnancy was associated with increased OXTR mRNA expression in all ages of mice compared to non‐pregnant myometrium; OXTR copy number increased 2.1‐fold in 3‐month‐old (*P < *0.001, *n = *8 for both groups), 2.4‐fold in 5‐month‐old (*P < *0.001, *n = *8 for both groups) and 1.8‐fold in 8‐month‐old (*P < *0.01, *n = *8 for both groups) late pregnant myometrium (Fig. 6 *B*).

### Spontaneous contractile activity of non‐pregnant and late pregnant myometrium

**Figure 7:**
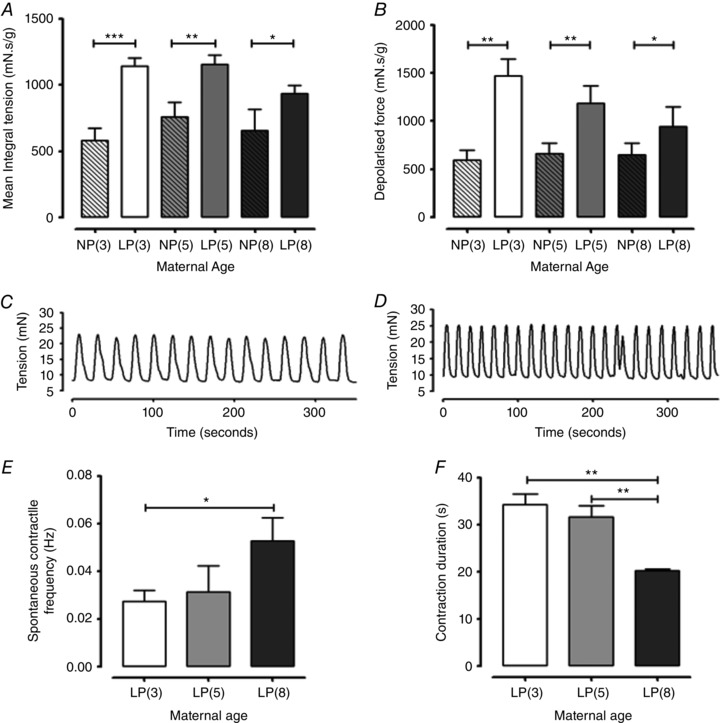
Effect of maternal age on spontaneous contractile activity in the myometrium from non‐pregnant and late pregnant mice Myometrium was obtained from non‐pregnant 3‐month‐old [NP(3), *n = *32 strips from *n = *8, open bars with diagonal lines], late pregnant 3‐month‐old [LP(3), *n = *32 strips from *n = *8, open bars], non‐pregnant 5‐month‐old [NP(5), *n = *32 strips from *n = *8, closed grey bar with diagonal lines], late pregnant 5‐month‐old [LP(5), *n = *32 strips from *n = *8, closed grey bar], non‐pregnant 8‐month‐old [NP(8), *n = *32 strips from *n = *8, closed dark grey bar with diagonal lines] and late pregnant 8‐month‐old [LP(8), *n = *32 strips from *n = *8, closed dark grey bar] mice for (*A*), (*B*), (*E*) and (*F*). *A*, MIT was significantly higher in late pregnant myometrium compared to non‐pregnant myometrium across all age groups (^***^ *P < *0.001, ^**^ *P < *0.01, ^*^ *P < *0.05). *B*, force amplitude was significantly higher in late pregnant myometrium compared to non‐pregnant myometrium across all age groups (^**^ *P < *0.01, ^*^ *P < *0.05). *C*, representative trace showing spontaneous myometrial contractions from a late pregnant 3‐month‐old mouse. *D*, representative trace showing spontaneous myometrial contractions from a late pregnant 8‐month‐old mouse. *E*, spontaneous myometrial contraction frequency was significantly greater in late pregnant 8‐month‐old *vs*. late pregnant 3‐month‐old mice (^*^ *P < *0.05). *F*, the myometrial spontaneous contraction duration was shorter in late pregnant 8‐month‐old *vs*. late pregnant 5‐month‐old and 3‐month‐old mice (^**^ *P < *0.01). Data were analysed using ANOVA followed by an all pairwise multiple comparison Tukey's test and are presented as the mean* ± *SEM for (*A*), (*B*), (*E*) and (*F*); all significant comparisons (*P* < 0.05) comparisons are indicated.

MIT values for spontaneous myometrial contractions were similar between all age groups in both non‐pregnant and late pregnant cohorts (*n = *8 mice per group, average of four tissue strips per animal) (Fig. 7 *A*). Spontaneous contractile activity was significantly higher in all late pregnant mice compared to non‐pregnant mice of all age groups (*P < *0.001, *P < *0.01, *P < *0.05, *n = *8 mice per group, average of four strips per animal).

The contractile response (mean peak amplitude) induced by 40 mm K^+^ PSS) was similar in the myometrium from all non‐pregnant age groups. In the late pregnant mouse, the numerical age‐associated reduction in the contraction amplitude was not significant (*P = *0.073) (Fig. 7 *B*). High K^+^‐induced contraction amplitudes were significantly increased in the myometrium from late pregnant mice compared to tissues from non‐pregnant mice in all three age categories (*P < *0.01, *P < *0.05, *n = *8 mice for each group, average of four tissue strips per animal) (Fig. 7 *B*).

The frequency and duration of spontaneous contraction in the myometrium from non‐pregnant mice was similar regardless of age. By contrast, spontaneous contraction was more frequent (1.7 times) in the myometrium from late pregnant 8‐month‐old mice (0.05 Hz, *n = *8 mice, average of four tissue strips per animal) compared to 3‐month‐old mice (0.03 Hz; *P < *0.05, *n = *8 mice, average of four tissue strips per animal) (Fig. 7 *C*–*E*).

The individual contraction duration in tissues from late pregnant mice aged 8 months was reduced by 36% and 41% compared to 5‐month‐old and 3‐month‐old myometrium, respectively (*P < *0.01 for both *n = *8 mice for all groups, average of four tissue strips per animal). No statistical difference was identified between mean contraction duration of myometrium from 3‐ *vs*. 5‐month‐old mice (Fig. 7 *F*).

### Effect of oxytocin on spontaneous contractions of non‐pregnant and late pregnant myometrium

In non‐pregnant mice, oxytocin (10^−12^ to 10^−7^ m) did not augment contractile activity of spontaneously contracting myometrium *in vitro*. All statistical comparisons across all age groups were also non‐significant (*n = *8 mice per group, average of four tissue strips per animal).

**Figure 8:**
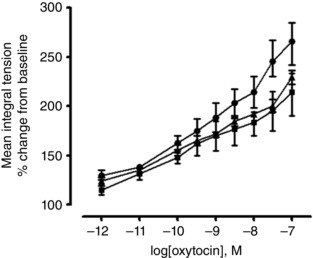
Effect of oxytocin (10^−12^ to 10^−7^ m) on the contractile activity in myometrial tissues taken from late pregnant mice at different ages The effect of oxytocin (10^−12^ to 10^−7^ m) on contractile activity in the myometrium from late pregnant 3‐month‐old [LP(3), *n = *32 strips from *n = *8, closed circles], late pregnant 5‐month‐old [LP(5), *n = *32 strips from *n = *8, closed triangles] and late pregnant 8‐month‐old [LP(8), *n = *32 strips from *n = *8, closed squares] mice. Data are expressed as the MIT, which is the percentage increase of the baseline spontaneous activity* ± *SEM. Application of oxytocin at concentrations 10^−12^ to 10^−7^ m was able to augment MIT in the myometrium from mice of all ages; linear regression analysis.

Oxytocin (10^−12^ to 10^−7^ m) augmented contractile activity (MIT) compared to baseline spontaneous contractile activity in late pregnant myometrium from mice in all age groups (Fig. 8). Compared to 3‐month‐old mice (slope 27.95, 95% CI = 23.41–32.49), the slope of the dose response was lower in 5‐month‐old (mean difference: –7.29, 95% CI = –12.31 to –2.28; *P < *0.01) and 8‐month‐old (mean difference: –7.63, 95% CI = –13.92 to –1.35; *P < *0.05) mice.

### mtDNA content in the myometrium

**Figure 9:**
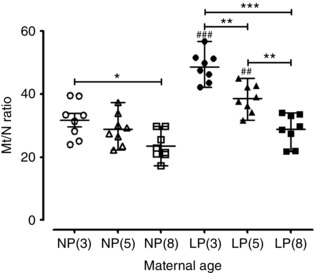
mtDNA copy number ratio (Mt/N) declines by the influence of maternal age the in non‐pregnant and late pregnant mouse myometrium Scatter graph depicting Mt/N in the myometrium from non‐pregnant 3‐month‐old [NP(3), *n = *8, open circles], non‐pregnant 5‐month‐old [NP(5), *n = *8, open triangles], non‐pregnant 8‐month‐old [NP(8), *n = *8, open squares], late pregnant 3‐month‐old [LP(3), *n = *8, closed circles], late pregnant 5‐month‐old [LP(5), *n = *8, closed triangles] and late pregnant 8‐month‐old [LP(8), *n = *8, closed squares] mice. Mt/N was significantly reduced by the influence of age between NP(3) and NP(8) myometrium (^*^ *P < *0.05), LP(3) and LP(8) myometrium (^***^ *P < *0.001), LP(3) and LP(5) myometrium (^**^ *P < *0.01) and LP(5) and LP(8) myometrium (^**^ *P < *0.01). Mt/N was increased by the influence of pregnancy between NP(3) and LP(3) myometrium (^###^ *P < *0.001) and NP(5) and LP(5) myometrium (^##^ *P < *0.01). Data were analysed using ANOVA followed by an all pairwise multiple comparison Tukey's test and are presented as the mean* ± *SEM.

The mtDNA copy number (Mt/N ratio) was 26% lower in the myometrium from non‐pregnant 8‐month‐old (23.56* ± *1.59 copy number) compared to non‐pregnant 3‐month‐old (31.75* ± *2.07; *P < *0.05) mice (Fig. 9). There was an age‐related decline in mtDNA copy number in late pregnant myometrium from 3 months (48.63* ± *1.67) to 5 months (38.61* ± *1.60; –25%; *P < *0.01) from 3 months to 8 months (28.80* ± *1.72; –41%; *P < *0.001) and from 5 months to 8 months (–21%; *P < *0.01) (Fig. 9). The mtDNA copy number was higher in late pregnant than non‐pregnant myometrium from 3‐month‐old (53%; *P < *0.001) and 5‐month‐old (34%; *P < *0.01) mice (Fig. 9).

### Mitochondrial electron transport chain enzyme activities in the myometrium

**Figure 10:**
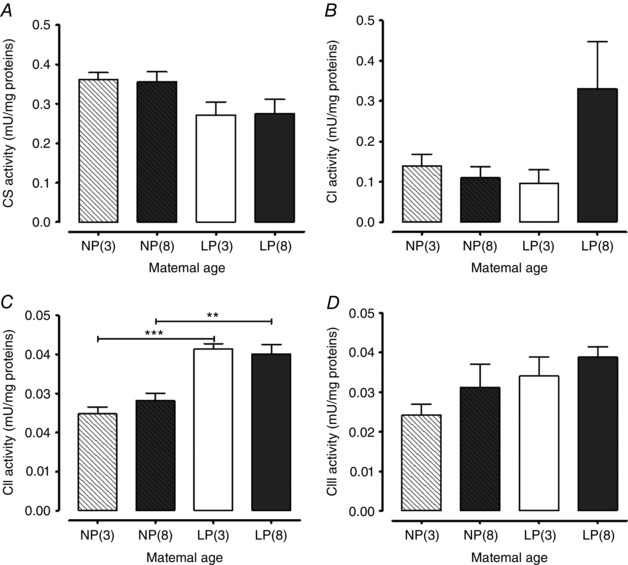
Effect of maternal age on citrate synthase, complex I, complex II and complex III enzymatic activities in the myometrium from non‐pregnant and late pregnant mice Myometrium was obtained from non‐pregnant 3‐month‐old [NP(3), *n = *8, open bar with diagonal lines], non‐pregnant 8‐month‐old [NP(8), *n = *8, closed dark grey bar with diagonal lines], late pregnant 3‐month‐old [LP(3), *n = *8, open bar] and late pregnant 8‐month‐old [LP(8), *n = *8, closed dark grey bar] mice for (*A*), (*B*), (*C*) and (*D*). *A*, there were no differences in citrate synthase enzyme activity. *B*, there were no differences in complex I/NADH dehydrogenase enzyme activity. *C*, transition from a non‐pregnant to pregnant state increased complex II/succinate dehydrogenase activity in the myometrium from both 3‐month‐old and 8‐month‐old mice (^***^ *P < *0.001, ^**^ *P < *0.01). *D*, there were no differences in complex III/ubiquinol cytochrome *c* reductase enzyme activity. Data were analysed using ANOVA followed by an all pairwise multiple comparison Tukey's test and are presented as the mean* ± *SEM.

Figure 10 *A* demonstrates that citrate synthase activity in myometrial tissue protein was not significantly affected by age in either non‐pregnant (3 *vs*. 8 months; *P = *0.896, *n = *8 for both groups) or late pregnant mice (3 *vs*. 8 months; *P = *0.952, *n = *8 for both groups). Citrate synthase enzyme activity was numerically similar in late pregnant mouse myometrium compared to the non‐pregnant state for both age sets; the differences were not significant (*n = *8 for all groups) (Fig. 10 *A*).

In tissues from the same groups of animals, complex I (NADH dehydrogenase) activity in myometrial protein was not modified by age in either non‐pregnant (3‐ *vs*. 8‐month‐old; *P = *0.465) or late pregnant mice (3‐ *vs*. 8‐month‐old; *P = *0.075) (Fig. 10 *B*). Myometrial tissue NADH dehydrogenase activity was not significantly altered by pregnancy in either age group compared to the non‐pregnant state (Fig. 10 *B*).

Similarly, complex II (succinate dehydrogenase) activity was not significantly altered by age in both non‐pregnant (3 *vs*. 8 months; *P = *0.205) and late pregnant mice (3 *vs*. 8 months; *P = *0.654) (Fig. 10 *C*). However, succinate dehydrogenase activity was significantly increased by pregnancy in the myometrium from both 3‐ and 8‐month‐old mice (*P < *0.001, *P < *0.01, respectively) (Fig. 10 *C*). Succinate dehydrogenase activity was significantly higher in the myometrium from pregnant 3‐month‐old (64%) and 8‐month‐old (43%) mice compared to age‐matched non‐pregnant controls (*P < *0.001 and 0.01, respectively).

Complex III (ubiquinol cytochrome *c* reductase) activity also was not significantly altered by age in non‐pregnant (3 *vs*. 8 months; *P = *0.299) and late pregnant mice (3 *vs*. 8 months; *P = *0.399) (Fig. 10 *D*). For both age groups, the enzyme activity in the myometrium was numerically higher in myometrial tissues from pregnant mice compared to the non‐pregnant state, although this did not reach significance (Fig. 10 *D*).

## Discussion

The present study confirmed the validity of using 8‐month‐old pregnant C57BL/6J mice, a common transgenic mouse strain, as a model of reproductive ageing in relation to parturition by demonstrating that maternal age was associated not only with prolonged gestation, but also greater duration of labour. Mechanistically, this was associated with an absence of progesterone withdrawal at term in older mice and disrupted uterine priming activation, although the process of cervical softening appeared to be unaffected. There was also a significant reduction in mtDNA copy number in the myometrium from older mice, which could contribute to impaired contractile activity via a reduction in overall ATP synthesis or altered intracellular Ca^2+^ regulation

### The impact of mouse maternal age on gestation length and labour duration

Accurate evaluation of timing of birth demonstrated that the older the age of mice at conception, the greater the probability of prolonged pregnancies and longer labours compared to younger mice. This supports previous data reporting a marked increase in gestation length seen with advancing maternal age across various species including mice and humans (Asdell, 1929; Terrill & Hazel, 1947; Brakel *et al*. 1952; Soderwall *et al*. 1960; Moore, 1963; Holinka *et al*. 1978; Silk *et al*. 1993; Jolly *et al*. 2000; Roos *et al*. 2010). The only previous study of aged C57BL/6J mice used proven breeders, rather than virgin mice, up to the age of 11–12 months and reported a gestation increase of 2.2 days (Holinka *et al*. 1978). Our data show that the length of gestation in primiparous C57BL/6J mice is prolonged from 8 months, which is an age that is more relevant to the human situation.

Labour duration was also significantly lengthened in older mice; previous animal studies have not examined this in any detail, as far as can be determined. Our data agree, however, with observations on reproductive ageing in humans where the length of labour and risk of prolonged labour increases with advancing maternal age (Greenberg *et al*. 2007). There are a number of possible explanations for the maternal age‐related prolonged gestation length in the mouse model, although the most probable is the observed absence of an effective progesterone withdrawal, which is essential for the onset of parturition in rodents (Weiss, 2000). Young animals showed a reduction in progesterone (72%) the day before delivery; in contrast, older animals maintained progesterone concentrations the day before delivery (only 28% reduction). Of interest, despite the lack of progesterone withdrawal, the older mice still went into labour. This brings into question the role of progesterone withdrawal in labour onset, although we cannot rule out the possibility that progesterone withdrawal occurs later and more quickly in older mothers and was not detected by our daily measurements up to 19 days. The apparent lack of progesterone withdrawal could also represent a disruption in the luteolysis pathway that inhibits/delays the progesterone decline in older mice. This was not assessed in the present study, although it has been reported that a significant decline in murine corpus luteum function during pregnancy normally only occurs at 10–11 months of age in mice (Harman & Talbert, 1970). One study of the effect of age on maternal progesterone concentrations in term parturient rats aged 8 and 24 weeks showed no difference, although this model was not one of advanced reproductive age (Elmes *et al*. 2015).

We also identified a negative relationship between reproductive age at conception and litter size at term. Older mice delivered smaller litters and had a greater incidence of stillbirth, with a small number of older mice failing to deliver by 22 days of gestation, most probably as a result of pup resorption. A reduction in litter size in older animals has been reported previously in various species (Soderwall *et al*. 1960; Finn, 1963; Holinka *et al*. 1978, 1979; Lopes *et al*. 2009; Monclus *et al*. 2011; Kong *et al*. 2012). Increased rates of resorption of conceptuses and intrauterine fetal deaths, as well as stillbirth rates, have also been reported in ageing rodents (Talbert, 1971; Holinka *et al*. 1979; Niggeschulze & Kast, 1994; Akiyama *et al*. 2006; Lopes *et al*. 2009; Kong *et al*. 2012).

The reduction in litter size in older mothers in the present study supports data from humans indicating that miscarriage increases with maternal age (Nybo Andersen *et al*. 2000; Nagaoka *et al*. 2011; Khalil *et al*. 2013; Qiao *et al*. 2014). Stillbirth and fetal loss are also common risk factors for mothers of advanced maternal age (Haavaldsen *et al*., 2010). Possible causes for fetal loss in older animals in the present study could be a series of reproductive senescence changes such as oocyte depletion and poor oocyte quality in aged ovaries resulting in the development of fewer and/or non‐viable fetuses (Liu *et al*. 2013). Decreased litter size can also be related to an increase in the number of defective blastocysts (as a consequence of abnormal embryonic development patterns) available for implantation (Day *et al*. 1991) or a failure of the aged uterus to maintain embryonic development (Talbert, 1971; Holinka *et al*. 1979; Kong *et al*. 2012). Furthermore, because 8‐month‐old mice tended to be slightly heavier, there may have been some influence of increased adiposity, although this was not controlled for in the present study. Unlike the study by Holinka *et al*. (1978) conducted in older animals, we did not find a significant relationship between litter size and gestation length. However, because this was not the primary focus of the present study, the numbers were small; it is possible that this relationship would become more apparent if more animals had been studied.

### The impact of mouse maternal age on cervical function

Having demonstrated that older mice have prolonged gestation and labour in parallel with altered progesterone withdrawal, we determined how this impacted on reproductive tissues and mechanisms underpinning the observed age‐related phenotypic changes. First, we hypothesized that delayed cervical softening (which occurs prior to ripening) in older mice could influence gestation length. Pregnant cervices were generally more distensible than those of non‐pregnant mice, comprising data that complements the existing body of literature in rodents (Harkness & Harkness, 1959; Read *et al*. 2007; Word *et al*. 2007; Vargis *et al*. 2012). Cervices from older pregnant mice still displayed greater distensibility than the younger pregnant mice but, intriguingly, this did not result in shorter labours. It suggests, perhaps, that the acute cervical ripening phase may have more functional importance for labour progress than cervical softening. We attempted to examine the contribution of collagen and MMP2 expression (Stygar *et al*. 2002; van Engelen *et al*. 2008; Choi *et al*. 2009; Akins *et al*. 2011) to these changes in cervix biomechanics and softening using immunolocalization techniques. There were no apparent age‐related changes in collagen and MMP2 in non‐pregnant mouse tissues. This did not mirror the generalized age‐associated changes reported for other collagen rich tissues and organs such as skin and bone (Wang *et al*. 2002; Quan *et al*. 2010).

In pregnant compared to non‐pregnant animals, there was a reduction in cervical tissue collagen content, which could directly account for the pregnancy related change in cervical distension. There were no significant differences in collagen and MMP2 expression in cervices on day 18 of gestation between younger and older animals. However, the period for cervical softening is long (days 12–18) and so differences may not be that apparent when labour is delayed. Indeed, the role of MMPs has been attributed more to the breakdown of the extracellular matrix during the ripening rather than the softening phase of parturition (Read *et al*. 2007). Because ripening usually occurs within 24 h of parturition (Read *et al*. 2007), this may potentially explain why no significant differences between MMP2 expression in non‐pregnant and late pregnant cervical tissues were detected. Cervical ripening also requires a drop in progesterone levels (Mahendroo *et al*. 1999; Mahendroo, 2012) and, because circulating progesterone remains relatively stable at the end of gestation in older 8‐month‐old mice, this may be an indication that the onset of cervical ripening was also perturbed.

Taken together, it is difficult to conclude that age‐induced changes in collagen and MMP2 cervical properties and cervical distension have a major impact on the progress of labour in this model. To confirm this definitively, further quantitative methods for comparing soluble and interstitial collagen content should be performed, along with western blotting and zymography for MMP2. Also the ∼24 h delay in parturition seen in 8‐month‐old mice needs to be taken into account by examining cervices on day 19 for this group.

### The impact of mouse maternal age on uterine function

We also determined that there was an inherent decrease in myometrial contractile function *in vitro* that could explain the prolongation of pregnancy and labour in older mice. These data provide additional insight into previous literature where a negative correlation between contractile activity and increasing maternal age has been reported for women (Smith *et al*. 2008) and in rats where mature parturient females demonstrate reduced spontaneous myometrial contractile activity compared to adolescent rats (Elmes *et al*. 2015).

At the molecular level, we detected small changes in Cx43 and OXTR expression in the older group of mice in parallel with the alterations in contractile frequency and duration in the myometrium. This suppression may be a result of the lack of progesterone withdrawal on day 18 in the older mice, or reduced fetal occupancy (reduced litter size) limiting the influence of uterine stretch on the induction of these contractile associated proteins (Ou *et al*. 1997; Ou *et al*. 1998; Lyall *et al*. 2002; Terzidou *et al*. 2005). This contrasts with the findings of Elmes *et al*. (2015) who reported that maternal age had no influence on Cx43 protein expression in pregnant rat myometrium but, as discussed previously, their previous study used 24‐week‐old rats, which represents full maturity rather than the point of reproductive decline as used in our model (Elmes *et al*. 2015). The upregulation of Cx43 and OXTR in late pregnancy compared to the non‐pregnant state was similar to other studies in rodents (Ou *et al*. 1997; Ou *et al*. 1998).

The functional impact of Cx43 and OXTR changes are difficult to determine directly because expression was still relatively high in older animals. However, assuming that the majority of the mRNA signal comes from smooth muscle rather than embedded blood vessels, this small decrease in mRNA could result in a reduction in gap junction number and OXTRs in the myometrium and partly explain the reduction in oxytocin‐augmented contractility in tissues from older mice. These data are consistent with previous findings that report reduced contractile responses to oxytocin in the myometrium from pregnant women aged 40 years and above (Arrowsmith *et al*. 2012).

In the present study, there was no specific age‐induced decrease in spontaneous contractile activity of non‐pregnant mice and oxytocin did not augment contractile activity, probably because OXTR mRNA expression was low. By contrast, Arrowsmith *et al*. (2012) have reported a striking reduction in spontaneous contractile force in non‐pregnant human myometrium from older women (post 30 years old) compared to myometrium from non‐pregnant 25–29‐year‐old women.

Returning to molecular events, despite evidence to support the role of PTGS2 in human and mouse pregnancy (Reese *et al*. 1999; Slater *et al*. 1999; Gross *et al*. 2000; Tsuboi *et al*. 2000; Tsuboi *et al*. 2003), the expression of PTGS2 was not induced in term myometrium or regulated by age. This might be because we assessed the myometrium on the day before delivery for the younger mice and 2 days before in the 8‐month‐old old group. Tsuboi *et al*. (2000) reported an absence of PTGS2 mRNA upregulation in the myometrium of wild‐type mice on the day before parturition but strong signals for PTGS2 mRNA on the day of parturition. Because myometrial tissues were taken on day 18 of gestation, it would be beneficial to repeat the present study using myometrial tissues collected on day 19 of gestation in older primiparous mice, as well as on the day of parturition for both young and older mice.

### The impact of mouse maternal age on uterine mitochondrial number and function

The unique set of experiments that we undertook to assess mitochondrial number and activity in the myometrium from primiparous mice in the early stages of reproductive ageing showed a clear reduction in the Mt/N DNA copy number seen with age in myometrial tissues from pregnant and non‐pregnant animals. Interestingly, there was a pregnancy‐associated enhancement in the Mt/N in younger mice, although this was suppressed in older mice. By contrast, mean activities of mitochondrial electron transport chain enzymes NADH dehydrogenase and ubiquinol cytochrome *c* reductase (complexes I and III), as well as citrate synthase activity, in the mouse myometrium were not influenced by age or pregnancy state. Succinate dehydrogenase (complex II) activity was also unaffected by age but was enhanced in pregnancy.

These data differ from the bulk of published data for cardiac and skeletal muscles and, to some extent, smooth muscle, where the process of ageing has often been associated with an alteration in the enzymatic activities of all or some of the mitochondrial electron transport chain complexes (Yen *et al*. 1989; Cooper *et al*. 1992; Torii *et al*. 1992; Sugiyama *et al*. 1993; Takasawa *et al*. 1993; Ojaimi *et al*. 1999; Kwong & Sohal, 2000; Lin *et al*. 2000; Short *et al*. 2005; Figueiredo *et al*. 2008; Muller *et al*. 2010; Padrao *et al*. 2012). Some contradictory data do exist, with alterations in mitochondrial respiratory complex activities not always being apparent across all tissues in aged animals (Kwong and Sohal, 2000).

The reasons for minimal age‐related changes in the present study may relate to the age of mice used because the majority of published studies have reported alterations in mitochondrial electron transport chain complex enzyme activities in muscles from animals far older than 8 months of age.

Irrespective of age, complex II was found to be significantly increased in the pregnant myometrium. This novel finding could be explained, in part, by an increased cell number and/or hypertrophy in the pregnant uterus (Douglas *et al*. 1988; Shynlova *et al*. 2006). However, we would also expect to see an increase in activities of all other electron transport complexes, which was not the case. Increased complex II activity is often associated with increased rates of reactive oxygen species (ROS) production by reverse electron flux from complex II to complex I (Quinlan *et al*. 2012; Dedkova *et al*. 2013). Increased ROS production leads to an eventual reduction of oxidative capacities and the ATP synthesis rate, which may be why the activities of complex I and III were not increased during pregnancy. To definitively determine mitochondrial function in terms of oxidative capacity and ATP production, NADH availability (NADH/NAD^+^ ratio) in the mitochondrial matrix should be measured.

The impact of pregnancy on cytochrome *c* oxidase/complex IV activity has been reported previously to increase in pregnant human and rat myometrium (Geyer & Riebschläger, 1974) compared to non‐pregnant myometrium, but the ages of women were not reported and the pregnant rats were only 40 days old.

The generalized age‐related reduction in mt/N DNA copy number in non‐pregnant and pregnant myometrium at 8 months of age suggests that this reduction precedes any change in mitochondrial complex activity. A reduced mitochondrial copy number has been reported previously as a consequence of ageing in rodent and human skeletal muscle (Barazzoni *et al*. 2000; Short *et al*. 2005; Peterson *et al*. 2012). According to the mitochondrial theory of ageing, a decline in mtDNA is a direct result of mitochondrial ROS accumulation, which in turn causes progressive damage to mtDNA and other mitochondrial constituents. The limited increase in mitochondrial copy number in pregnant older animals compared to non‐pregnant animals probably has physiological consequences in parturition and could help explain the reduction in contractile potential in the myometrium from older mice. A reduction in Mt/N could reflect a concurrent reduction in capacity with respect to generating sufficient cellular energy (ATP) to drive uterine contractions but, because several copies of mtDNA can be present in one mitochondria (Wiesner *et al*. 1992), ATP production in mitochondria from older animals would have to be measured to confirm this.

The role of mitochondria and ATP provision in the control of uterine contractions has been studied previously in the mouse non‐pregnant myometrium (Gravina *et al*. 2010). Pharmacological inhibition of mitochondrial complex I and disruption of the mitochondrial membrane potential caused a reduction in the force of myometrial contractions *in vitro*. It was hypothesized that these reductions were not linked to ATP generation but suggested instead that mitochondrial Ca^2+^ handling has an independent role in determining uterine contractility (Chalmers & McCarron, 2008; Gravina *et al*. 2010; Gravina *et al*. 2011). This could comprise an alternative explanation (given the reduction in mtDNA copy number) for our observations showing that myometrium from older pregnant mice has altered contractility *in vitro*.

### Summary

The present study confirms that both gestation and parturition are prolonged in a primiparous mouse model of maternal ageing, and there also is deterioration in reproductive capacity as reflected by a decline in litter size. The mouse model mimics the characteristics associated with ageing in humans, including a delayed onset of parturition and a prolonged duration of labour, as well as a greater risk of fetal loss. It therefore comprises a suitable model for assessing further the mechanisms responsible for the age‐induced changes demonstrated in the present study.

The present study demonstrates that maternal age influences the expression of contractile associated proteins and also that alterations in spontaneous and oxytocin augmented myometrial contractions in older mice. These data suggest that myometrium from older mice on day 18 of gestation may not be fully ‘activated’ for parturition mediated either by the limited progesterone withdrawal in the older mice or a reduced uterine stretch as a result of litter size. A reduced Mt/N copy number in myometrial tissues from older pregnant mice implies that myometrium in older mice have fewer mitochondria, with a potential impact on myometrial contractile function in these animals.

Taken together, the data from the present study highlight the physiological and cellular changes that occur with reproductive ageing. The reproductive tissue that is predominantly affected by ageing is the myometrium. Our work highlights the need for additional research into this nascent area in both animal models and human tissues, with the aim of developing a more detailed understanding of the mechanistic impact of maternal ageing on labour. This is necessary to the inform management of labour in older women, which is a burgeoning clinical problem.

## Additional information

### Competing interests

The authors declare that they have no competing interests.

### Author contributions

RP, RMT, JDM, EM, LD and LP designed the experiments. RP performed most of the experiments at King's College London, UK. Immunohistochemistry was carried out by RP and JDM at St George's University of London, UK. Experiments to measure mitochondrial electron transport chain enzymatic activities were performed by RP and EM at Université Joseph Fourier, Grenoble, France. RP, EM and PTS analysed and interpreted data. RP, RMT, JDM, EM, LD, PTS and LP wrote the manuscript and critically revised it before submission. All authors approved the final version of the manuscript submitted for publication.

### Funding

The present study was supported by a BBSRC PhD studentship (BB/G017190/1) and Tommy's charity (No. 1060508).
